# Sentiment analysis of post-COVID-19 health information needs of autism spectrum disorder community: insights from social media discussions

**DOI:** 10.3389/fpsyt.2024.1441349

**Published:** 2024-10-11

**Authors:** Ebenezer Larnyo, Jonathan Aseye Nutakor, Stephen Addai-Dansoh, Edmund Nana Kwame Nkrumah

**Affiliations:** ^1^ Center for Black Studies Research, University of California, Santa Barbara, Santa Barbara, CA, United States; ^2^ Department of Health Policy and Management, School of Management, Jiangsu University, Zhenjiang, Jiangsu, China; ^3^ Nobel International Business School, Accra, Ghana

**Keywords:** autism spectrum disorder, health information needs, social media analysis, sentiment analysis, community support, technological solutions, post-COVID-19

## Abstract

**Objective:**

This study explores the health information needs of individuals with autism spectrum disorder (ASD) and their caregivers in the post-COVID-19 era by analyzing discussions from Reddit, a popular social media platform.

**Methods:**

Utilizing a mixed-method approach that integrates qualitative content analysis with quantitative sentiment analysis, we analyzed user-generated content from the “r/autism” subreddit to identify recurring themes and sentiments.

**Results:**

The qualitative analysis uncovered key themes, including symptoms, diagnostic challenges, caregiver experiences, treatment options, and stigma, reflecting the diverse concerns within the ASD community. The quantitative sentiment analysis revealed a predominance of positive sentiment across discussions, although significant instances of neutral and negative sentiments were also present, indicating varied experiences and perspectives among community members. Among the machine learning models used for sentiment classification, the Bi-directional Long Short-Term Memory (Bi-LSTM) model achieved the highest performance, demonstrating a validation accuracy of 95.74%.

**Conclusions:**

The findings highlight the need for improved digital platforms and community resources to address the specific health information needs of the ASD community, particularly in enhancing access to reliable information and fostering supportive environments. These insights can guide future interventions and policies aimed at improving the well-being of autistic persons and their caregivers.

## Introduction

The Autism Spectrum Disorder (ASD) community constitutes a diverse group of individuals with unique needs and challenges. Navigating life as an autistic person can be a complex and demanding journey, encompassing various aspects of health and well-being (1, 2). For autistic individuals, access to relevant and comprehensive health information is not only essential but can significantly impact their overall quality of life (3, 4). In the wake of the COVID-19 pandemic, the importance of addressing the specific health information needs of this population has become even more pronounced (5).

ASD is a neurodevelopmental condition characterized by differences in social communication, behavior, and sensory processing (6, 7). Autistic individuals may have co-occurring health conditions, from mental health concerns like anxiety and depression to gastrointestinal problems and sleep disorders (8, 9). The complex interplay between these health issues and the core characteristics of ASD necessitates tailored healthcare approaches and targeted information dissemination (10, 11).

Historically, obtaining a nuanced understanding of the health information needs of autistic individuals has been challenging (12–14). The limited availability of high-quality data and research on this topic has impeded the development of effective strategies for addressing these needs. However, in recent years, the explosion of social media platforms has created new opportunities to gain insights into the daily lives, concerns, and information-seeking behaviors of people with ASD.

This study seeks to leverage social media, specifically Reddit, as a valuable source of data to uncover and gain insight into the health information needs of autistic individuals and their families, with a focus on the post-COVID-19 era. Reddit, as one of the most popular online forums, provides a unique platform where autistic individuals and their caregivers openly discuss their experiences, challenges, and questions related to health and well-being. By analyzing the content of these discussions, we aim to bridge the existing knowledge gap and shed light on the specific health information needs of this population in a post-pandemic world.

### The impact of COVID-19 on autistic individuals

The COVID-19 pandemic, caused by the novel coronavirus SARS-CoV-2, brought unprecedented challenges and disruptions to the lives of individuals worldwide (15). For autistic individuals, who often thrive on routine and predictability, the sudden changes brought about by lockdowns, school closures, and social distancing measures posed unique difficulties (16, 17). The pandemic has disrupted access to vital support services, including therapy, education, and social activities, leading to heightened stress and anxiety for autistic persons and their families (18–20). Moreover, the pandemic’s impact on the healthcare system has been profound. Hospitals and clinics were overwhelmed with COVID-19 cases, diverting attention and resources away from routine medical care and specialized services for autistic persons (19, 21). This has exacerbated existing healthcare disparities and created additional barriers to accessing appropriate care. Also, autistic persons’ reliance on routine, difficulty with change, and comprehending social cues, left them vulnerable to the disruptions caused by lockdowns, social distancing, and remote services (22, 23). For instance, they experienced disruption in the everyday pattern which was already difficult for them as they depend on predictability to navigate society (23). Additionally, school closures, canceled therapy appointments, and altered family routines led to increased anxiety and behavioral challenges for autistic individuals. The lack of in-person social interactions exacerbated difficulties in communication and social skills development, which are key symptoms of ASD (24).

As nations move into the post-COVID-19 phase, it is imperative to understand how this unprecedented event has affected the health information needs of autistic persons. The experiences of individuals on the spectrum, their caregivers, and the healthcare providers who serve them during the pandemic have undoubtedly shaped their concerns and priorities regarding health and well-being. By examining social media data from the post-COVID-19 period, we aim to capture these evolving needs and provide insights that can inform healthcare strategies and policy decisions.

### The role of social media in understanding health information needs

Social media platforms have become invaluable sources of information and support for individuals facing health challenges (25–27). For instance, platforms like Twitter and Facebook have been widely used to study public health trends, such as the spread of infectious diseases (e.g., COVID-19) (28–30), mental health conditions, and chronic illnesses like diabetes and heart disease. Studies have shown that social media allows for real-time data collection, capturing immediate public reactions and concerns, which traditional methods may not achieve as efficiently (27, 31). One of the primary advantages of using social media for health research is the ability to access diverse, unfiltered patient perspectives and experiences. Unlike structured surveys or interviews, social media discussions are often more candid and spontaneous, providing richer qualitative data (26). This is particularly beneficial for understanding the nuanced experiences of individuals with complex health conditions like ASD, where traditional research methods might not fully capture the diversity of experiences and challenges faced by this community. For autistic individuals and their families, these platforms offer a sense of community, a space to share experiences, and a forum for seeking advice and information (32–34). Different social media platforms offer unique advantages. Platforms like Twitter, while effective for tracking public health trends such as the spread of infectious diseases like COVID-19 and monitoring mental health and chronic illnesses (35), are often less conducive to in-depth, sensitive discussions due to their lack of anonymity and character limits (26, 36). In contrast, Reddit offers a unique space where users can engage anonymously in community-driven discussions (37, 38), facilitating more open and honest exchanges, particularly on sensitive health topics like mental health (39) and autism spectrum disorder (ASD) (40). Furthermore, Reddit’s structure of “subreddits,” or topic-specific forums, allows users, including autistic individuals, caregivers, and healthcare professionals to participate in detailed conversations about the challenges they face and the health information they seek. This structure promotes richer qualitative data that might be missed on platforms like Twitter, where brevity and public visibility are limiting factors.

By analyzing discussions on autism-related subreddits, we can gain insights into the health information needs of the autistic community, capturing a wide range of perspectives in real-time.

## Methodology

### Data collection and preprocessing

To accomplish the objective of this study, a mixed-method approach that combines qualitative content analysis with quantitative data analysis techniques was employed. Reddit hosts numerous subreddits dedicated to autism and related topics, making it a valuable source of user-generated content. We identified and selected a set of subreddits that were focused on ASD, health, and well-being, or encompassed discussions that frequently touched upon these topics. Based on the assessment, the “r/autism” subreddit, which had the highest number [approximately three hundred three thousand (303,000)] of active members, was chosen to ensure a diverse range of perspectives and experiences within the ASD community. The data was collected between March and June 2023, specifically focusing on posts and comments made during the post-COVID-19 era, using Reddit’s Application Programming Interface (API). This timeframe was selected to capture the evolving health information needs of autistic individuals in the context of the pandemic’s impact. The data collection was driven by a set of predefined keywords based on an extant literature review, which identified 11 core themes: symptoms, care/treatment, drugs/interventions, epidemiology, stigma, diagnosis, assistive technologies (smart), burden of ASD, caregivers of ASD, natural cure, and COVID and ASD. Keywords such as “inequality,” “underdiagnosis,” “misdiagnosis,” “sensory issues,” “behavioral therapy,” “Risperidone,” “prevalence,” “social isolation,” “DSM-5,” “wearable technology,” “economic burden,” “parental stress,” “homeopathy,” “teletherapy,” and others were used to scrape relevant data. The selection of these keywords was intended to capture a broad spectrum of discussions that reflect the diverse experiences and needs of the ASD community. The collected data included title, selftext content, timestamps, url, author/user information (anonymized for privacy), and post/comment metadata (num_comments and upvotes). To avoid the overrepresentation of highly active users, only one post per user was randomly selected for analysis. This provides equal representation across the users and ensures a diverse range of voices are present within the dataset. Before analysis, we performed preprocessing to clean and prepare the data for analysis. This included removing irrelevant posts, duplicates, and spam, as well as anonymizing any personally identifiable information. The preprocessing phase also involved tokenizing the text data and padding the sequences to ensure uniform input size for all models. The Tokenizer class from TensorFlow was used to convert text into sequences of integers, representing the vocabulary index of each word. A maximum vocabulary size of 10,000 was set to limit the number of unique words, and sequences were padded to a length of 100 tokens using a function. Additionally, the labels (sentiment of the posts) were encoded using a label encoder to convert categorical labels into integers. The data was then split into training and test sets with an 80-20 split for the classification of sentiments. A total of 232 users and their respective posts were further analyzed after preprocessing the data.

### Data analysis

The qualitative content analysis focused on understanding the themes, patterns, and sentiments in the collected Reddit posts and comments. The cleaned data was processed to match the predefined keywords with the corresponding themes. This involved systematically reviewing each post to determine its relevance to the identified themes based on keyword presence and context. This thematic categorization allowed for a structured qualitative analysis, where posts were coded according to the identified themes. The coding process was iterative, refining the themes through repeated immersion in the data to ensure that the analysis accurately captured the community’s expressed needs and concerns.

In addition to qualitative content analysis, a quantitative method was employed to extract and analyze specific data points from the data. This was done to complement the qualitative findings and provide numerical insights into various aspects of the data, such as the frequency of themes, sentiment analysis, and correlation between sentiments and themes. The theme frequency analysis was used to identify the most commonly mentioned terms and phrases related to health information needs and concerns in the posts and comments. Sentiment analysis was then performed to assess the overall sentiment expressed in the ‘selftext’ and ‘title’. This analysis aimed to gauge whether discussions were predominantly positive, negative, or neutral in tone. Specific criteria were applied to the data to ensure the reliability and relevance of the results. Out of the 234 posts extracted, 232 users and their respective posts were included in the sentiment analysis. Posts were excluded if they had an empty or minimal ‘selftext’ and ‘title’ fields, contained excessive non-textual content, or could not be processed effectively due to formatting or content quality issues. These exclusions were necessary to focus the analysis on ‘selftext’ and ‘title’ with substantial textual content suitable for accurate sentiment classification. Five models; Bi-directional long short-term memory (Bi-LSTM) (41–43), convolutional neural network (CNN) (44–46), Distilled Bidirectional Encoder Representations from Transformers (DistilBERT) (47, 48), gated recurrent unit (GRU) (49–51), and ensemble model combining the predictions of the Bi-LSTM, CNN, and GRU models (52–55) were implemented to perform the sentiment classification. Each model was trained on the training dataset for five epochs. The validation dataset was used to evaluate model performance during training, allowing for the assessment of both training and validation loss and accuracy. The optimal model was selected as a natural language processing (NLP) method for the study.

### Ethical considerations

Respecting ethical guidelines and ensuring user privacy were paramount throughout this research. All collected data were anonymized to protect the identities of users. Additionally, no personally identifiable information or sensitive data were collected.

## Result

**Table 1** shows the frequency metric of the total occurrences of each theme across all posts analyzed. Each theme was counted independently, that is, a single post could contribute to multiple theme counts if it discussed more than one theme. The qualitative content analysis provided deeper insights into the context and nuances behind the frequency data, examining user posts in relation to key themes. Results of the qualitative analysis, including representative quotes, and thematic interpretation, are presented in **Supplementary Table 1**. The table highlights how specific quotes informed our conclusions and recommendations.

**Table 1:** Frequency of themes.

Theme	Frequency	Percentage
Symptoms	134	57.51%
Diagnosis	40	17.17%
Caregivers of ASD	29	12.50%
Drugs/interventions	7	3.02%
Natural cure	6	2.59%
Care/treatment	6	2.59%
Stigma	5	2.16%
Smart assistive technologies	4	1.72%
Burden of ASD	1	0.43%

Results from **Table 1** showed that symptoms emerged as the most dominant within the discussions, encompassing more than half of the dataset. The “diagnosis” theme, while less frequent than “symptoms,” remains a substantial topic of interest. Caregiver/caregiving also emerged as another prominent theme, highlighting the crucial role played by caregivers in the lives of autistic persons. The “drugs/interventions” theme signifies discussions on pharmacological treatments, therapeutic interventions, or medical approaches for managing ASD. While less frequent than themes such as symptoms, and diagnosis, these conversations are essential as they reflect the pursuit of effective interventions and therapies. Natural cures representing discussions about alternative or holistic approaches to addressing ASD and care/treatment, which focuses on aspects related to the care and treatment of autistic individuals, accounted for 2.16% of the posts analyzed.

**Figure 1:**
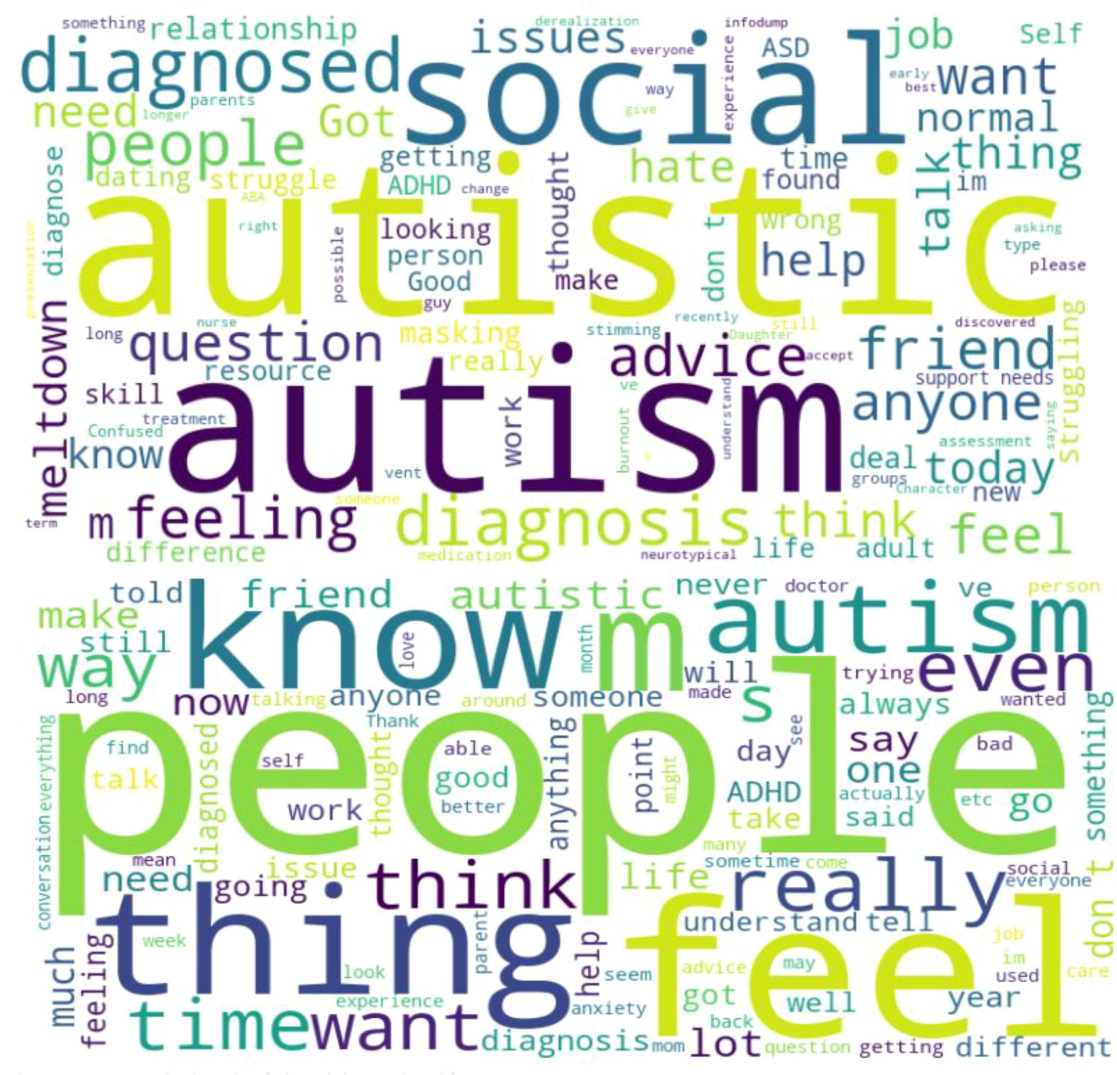
Word cloud of the title and self-text.

Themes such as stigma, smart assistive technologies, and the burden of ASD, which encompassed discussions about the emotional, financial, and societal burdens associated with ASD were all part of the frequent themes related to the discourse on Reddit. **Figure 1** shows the visualization result of the ‘self-text’. It appeared that the most frequent words were ‘people’, ‘know’, ‘autism’, feel’, and ‘thing’.

**Figure 2:**
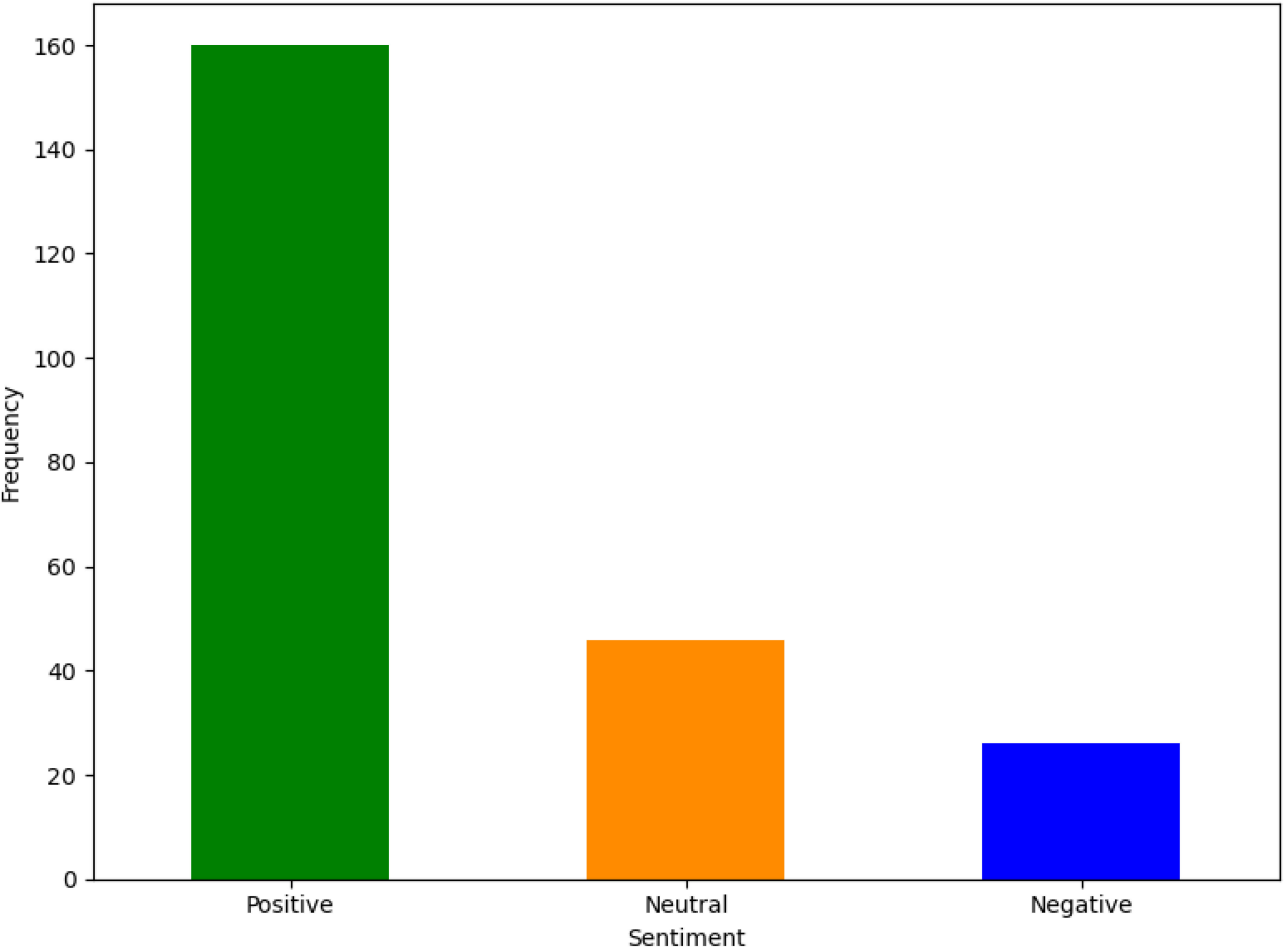
Result of overall sentiment analysis on the self-text and title.

**Figure 2** shows the visualized result of the sentiment analysis conducted on the 232 posts for the self-text and title. The sentiment categories; positive, negative, and neutral, were derived from an automated analysis of the textual content (self-text and title) of the posts.

**Figure 3:**
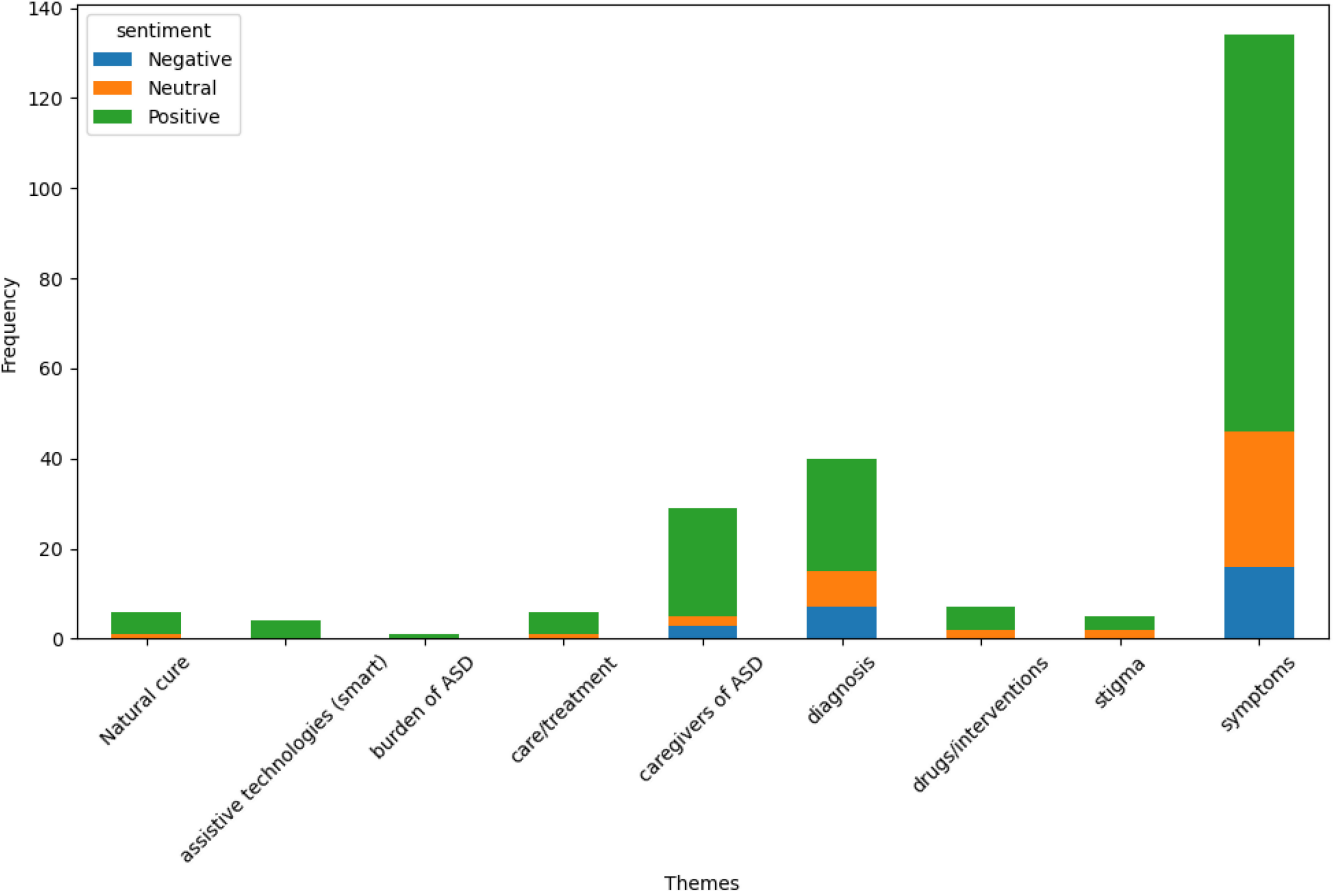
Correlation between themes and sentiments.

Based on the result, it was revealed that the majority of the posts (160 out of the total) expressed a positive sentiment, 26 expressed negative sentiments, and 46 were neutral sentiments. The negative sentiments encompassed expressions of dissatisfaction, criticism, or concerns regarding various themes. A correlation between different themes related to autism spectrum disorder (ASD) and the sentiment expressed in discussions on these themes was performed as shown in **Figure 3**. Symptoms had a significant correlation with the number of positive sentiments (88) and a significant number of neutral (30) and negative (16) sentiments. This suggests that discussions about symptoms are prevalent and diverse in the sentiment, potentially reflecting a wide range of experiences and perspectives. Diagnosis also had a considerable number of positive (25), neutral (8), and negative (7) sentiments, indicating mixed feelings in the discussions on this theme. The caregivers of ASD theme also revealed a high level of positive sentiment (24), with a few neutral (2) and negative (3) posts, suggesting supportive or empathetic discussions around caregivers.

In contrast, it was observed that themes like “Natural cure,” “assistive technologies (smart),” “burden of ASD,” and “stigma” have lower frequencies and are predominantly discussed with positive or neutral sentiments, indicating less controversy or a more optimistic view in these areas.

Results of the model training revealed that training loss and validation loss decrease progressively with each epoch, indicating that the model is learning and improving its performance on both the training and validation datasets. The training accuracy and validation accuracy were observed to remain high (around 0.96) throughout all epochs, suggesting that the model is performing well and there is no significant overfitting occurring.

**Table 2** summarizes the performance of the trained models on a held-out validation set for Bi-LSTM, CNN, GRU, DistilBERT, and Ensemble. Each model was trained for 5 epochs. For all models, the training accuracy remains constant at 0.9622 across all epochs, indicating that the models fit the training data well. The validation accuracy remains constant at 0.9574 across all epochs for all models, suggesting that the models are not overfitting to the training data and are generalizing well to unseen data. The training loss generally decreases with each epoch for all models, indicating that the models are learning and improving their performance on the training data.

**Table 2:** Performance metrics for the trained models.

Model	Epoch	Training Loss	Training Accuracy	Validation Loss	Validation Accuracy
Bi-LSTM	1	0.4171	0.9622	0.3791	0.9574
	2	0.2984	0.9622	0.2573	0.9574
	3	0.1998	0.9622	0.1845	0.9574
	4	0.1679	0.9622	0.1825	0.9574
	5	0.1707	0.9622	0.1795	0.9574
CNN	1	0.2367	0.9622	0.3271	0.9574
	2	0.2535	0.9622	0.3315	0.9574
	3	0.2248	0.9622	0.2957	0.9574
	4	0.1809	0.9622	0.2563	0.9574
	5	0.1506	0.9622	0.2208	0.9574
GRU	1	0.6922	0.9622	0.6556	0.9574
	2	0.5063	0.9622	0.3586	0.9574
	3	0.2144	0.9622	0.1884	0.9574
	4	0.1575	0.9622	0.2047	0.9574
	5	0.1786	0.9622	0.2025	0.9574
DistilBERT	1	15.5448	0.0270	0.4234	0.9574
	2	0.4757	0.9622	0.5403	0.9574
	3	0.4178	0.9622	0.4406	0.9574
	4	0.3388	0.9622	0.3134	0.9574
	5	0.2180	0.9622	0.2168	0.9574
Ensemble	1	0.1492	0.9622	0.1828	0.9574
	2	0.1392	0.9622	0.1910	0.9574
	3	0.1311	0.9622	0.1896	0.9574
	4	0.1167	0.9622	0.2008	0.9574
	5	0.1052	0.9622	0.2080	0.9574

**Figure 4:**
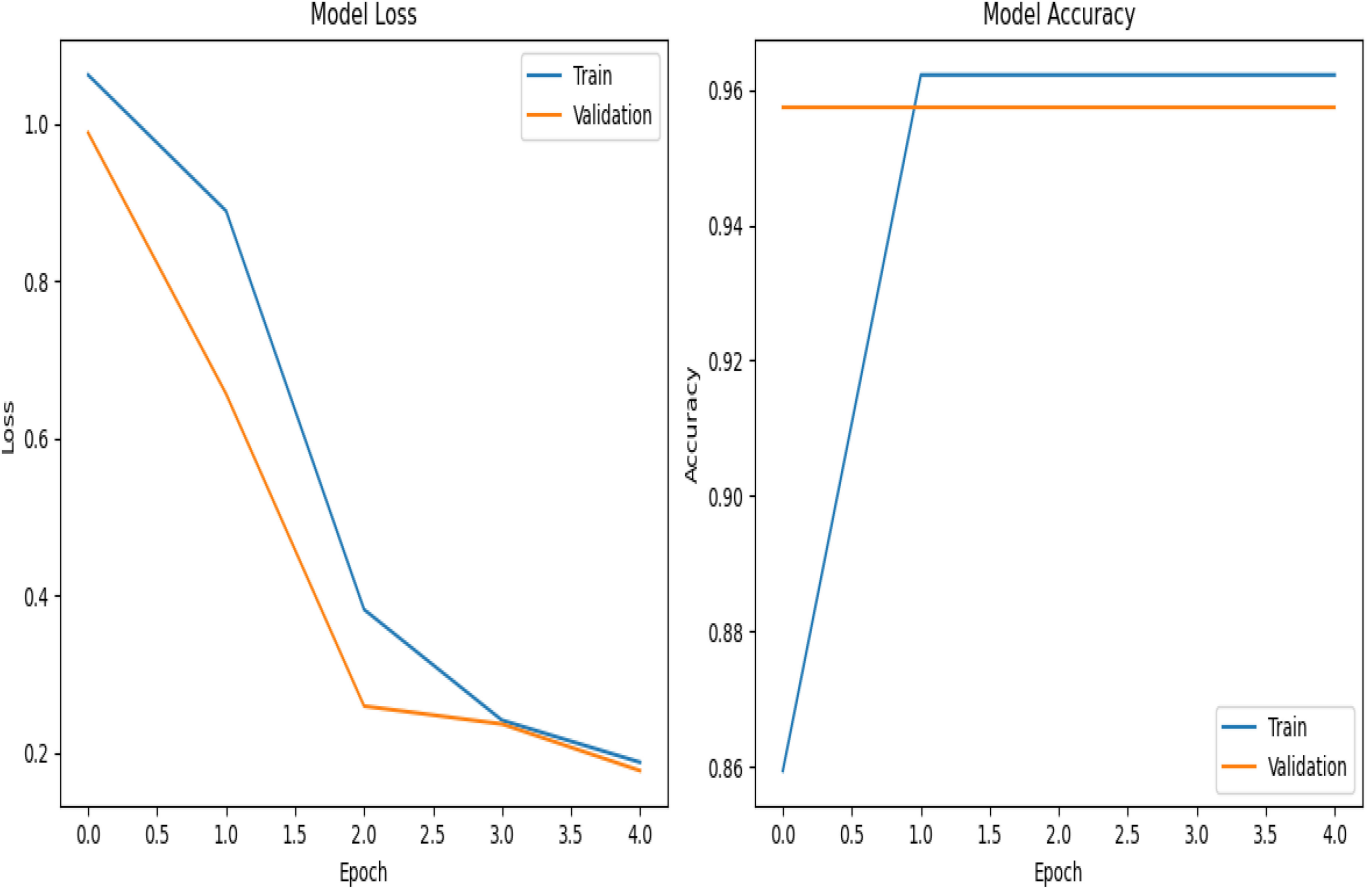
Bi-LSTM model.

Among the evaluated models, Bi-LSTM achieved the lowest validation loss (0.1795) while maintaining a high validation accuracy (95.74%), a pictorial representation is shown in **Figure 4**.

## Discussion

This study aims to investigate the health information needs of autistic persons and their caregivers post-COVID-19 and provide viable solutions by analyzing data scrapped from Reddit, a social media platform. The distribution of themes provides a comprehensive view of the topics that resonate most with the Reddit community discussing ASD. It also reflects the diverse concerns and interests within the community, ranging from symptom descriptions and diagnostic challenges to treatment options and the need to address stigma. Across the most frequently discussed topics, including symptoms, caregivers of ASD, and diagnosis, a number of recurring themes are present. Broadly, these themes fall into the following categories: (1) psychosocial impact of ASD and possible comorbid mental health disorders, and (2) diagnostic journey and professional support. Within the category of psychosocial impact and possible comorbid mental health disorders, individuals seek to share the struggles they encounter within interpersonal and intrapersonal relationships that result from their symptoms. This is done in order to feel understood and/or accepted and/or to receive guidance. Interpersonal relationships can range from familial, romantic, friendship, workplace, and societal norms/expectations. Intrapersonal struggles revolved around self-identity and acceptance, imposter syndrome and internal symptom regulation. The authors of these reddit posts are majority autistic persons but some do not have ASD but are in close proximity to one or more individuals who do. Many autistic individuals also disclosed that they have comorbid mental health disorders that further exacerbate the issues mentioned above. Excerpts from a Reddit user is shown below:

“*Hi. I’m quite new to all the neurodivergent stuff. I’m 20 and got my diagnosis (Autism with ADHD) roundabout a year ago.*”

“*I diagnosed myself with ADHD around the age of 25. My son was diagnosed with ADHD and HFA around 7. Both confirmed by several specialists.*”

“*But I’m also afraid it could be linked to my depression and/or autism.*”

Within the category of diagnostic journey and professional support, we observed that individuals mainly express the desire to obtain a diagnosis, share their reaction to receiving a diagnosis in order to feel understood and receive guidance, as well as share their concerns and experiences with healthcare professionals. It is important to note that the most imperative theme across the board is the resilience, desire, and determination of autistic persons to find fulfillment in life. It is of equal importance that professionals and scholars in the healthcare system do their due diligence to provide viable solutions to these individuals.

It is evident that technological solutions are relevant when discussing how to address the challenges faced by autistic individuals (56, 57). A proposed solution to these challenges is a mobile/tablet application that coincides with a website that provides the following: (1) everyday research-backed tools that are categorized by common issues autistic individuals encounter, (2) a (monitored) interactive aspect where autistic persons or their caregivers can communicate with one another and with healthcare professionals. Although not everyone has equal access to technology (58) and there needs to be an effort to increase accessibility, research shows that the majority of U.S. households have access to both broadband internet and either a smartphone, tablet or computer (57, 59–61). Given this finding, an app or website as described above would be a viable resource for autistic individuals, their caregivers, and communities.

Additionally, it is apparent that there is a necessity for interconnectedness in communities between autistic persons, ASD advocacy organizations, healthcare institutions and professionals, local libraries, community centers, academic institutions, and other relevant institutions (56, 62). This unified effort will ensure that communities are educated and aware of what signs of ASD may look like, where to find appropriate resources, and so that those with ASD feel understood, accepted, and supported by the communities they belong to. This also leads to streamlining that makes a clear, achievable pathway for autistic individuals to be able to easily seek out the necessary resources, receive a diagnosis, and gain tools that would mitigate the adverse effects caused by not knowing what to do, where to go, or be able to identify what the “issue” is. In conjunction with this effort, there needs to be an expansion and increased availability of healthcare institutions that can provide diagnostic services, taking into account which areas are underserved. This is crucial because the interconnectedness within a community is unachievable if the necessary institutions are present to begin with. This requires funding to be put towards establishing or improving accessible facilities within and near underserved areas.

Addressing the aforementioned issues that autistic persons and their caregivers face aligns with principles of social justice and equity in order to ensure all members of society have equal opportunities to lead fulfilling lives and aid these individuals in reaching their fullest potential and being valuable contributions to society. Furthermore, doing so not only benefits autistic individuals but also promotes overall well-being in communities as a whole (56). These findings can guide future research endeavors, community initiatives, and support services to better cater to the needs and interests of autistic persons and their families. Additionally, understanding the prevalence of these themes can facilitate the creation of targeted educational resources and awareness campaigns, ultimately contributing to a more informed and supportive community for autistic persons and their caregivers.

Finally, of the models used in this study; Bi-LSTM, CNN, GRU, DistilBERT, and Ensemble, Bi-LSTM yielded the best optimal parameters validation loss (0.1795) and a high validation accuracy of 95.74%.

## Conclusion

Using data from Reddit, this study provided insight into the health information needs of autistic individuals and their caregivers during COVID-19. Results of the study reveal that individuals with ASD and their caregivers engage in discussions that are centered on the psychosocial impact of autism spectrum disorder, the difficulties they encounter coupled with the complexities of their diagnostic journey, and the challenges they face in accessing professional support. The findings reiterate the need for understanding, guidance, and community support, as well as the need for tools and resources that can help militate against the adverse challenges associated with autism spectrum disorder. Based on the findings of this study, we propose the development of digital platforms i.e. mobile/tablet applications and websites, designed to offer reliable and interactive health information to this key population. Such platforms will not only address the technological and digital divide and curb misinformation but also foster a sense of community and interconnectedness, which is crucial for autistic persons to feel understood and supported. Furthermore, by expanding and improving access to affordable diagnostic services, particularly in impoverished communities, we will ensure that all persons, irrespective of their geographical location and socioeconomic status, have access to the resources they need to live a fulfilling life, thus reducing health disparity and promoting social justice and equity. This study also highlights the resilience and determination of autistic individuals and their caregivers, underscoring the importance of creating a supportive environment that enables them to excel. By addressing the gaps in the health information need and support, this work contributes to a more informed and inclusive approach to dealing with issues of ASD with implications for future research, community initiatives, targeted intervention, and policy development. Finally, the methodological technique adopted in this study, particularly the use of the Bi-LSTM model, which achieved high validation accuracy, provides a robust framework for analyzing social media data in health research.

### Limitations of the study

Though this study provides valuable insights into the health information needs of the ASD community post-COVID-19, it had some limitations. Since our data collection was confined to Reddit, it may not be representative of the entire ASD community. Users of Reddit might have different demographics, levels of engagement, and experiences compared to those who do not use social media or prefer other platforms. Hence, this potential selection bias could limit the generalizability of our findings to the broader ASD population. Furthermore, since only one post from the users was selected for analysis, this may give rise to selection bias. While this approach ensures equal representation amongst users, it may fail to portray more contextual discussions by highly active users. Hence, future research should consider analyzing multiple posts by a user to get a fuller range of user experiences. The data used for this study is based on self-reported information shared in Reddit posts and comments. This type of data could be subject to biases, such as social desirability bias, recall bias, and selective sharing of information, hence the accuracy and reliability of the data might be compromised, as users may present themselves in a more favorable light or omit critical details. As a result, the findings should be interpreted with caution, considering the inherent limitations of self-reported data. Our study focused on a specific timeframe (March to June 2023) and a single subreddit (“r/autism”). While this allowed for a manageable dataset and a focused analysis, it might not capture the full range of experiences and concerns of the ASD community over a longer period or across different subreddits. The findings might not reflect longer-term trends or the diversity of discussions occurring in other online forums, thus limiting the scope of our conclusions. Additionally, posts used for this study were in English. This introduces a language bias, potentially excluding non-English speakers. As a result, the insights gained may not apply to non-English-speaking ASD communities, limiting the global relevance of the findings. Lastly, though the data scrapping, cleaning, preprocessing, and analysis were rigorously carried out, the automated sentiment analysis and coding processes, might misinterpret the nuances and context of user discussions. Sentiment analysis tools can sometimes inaccurately classify sentiments, particularly in complex or ambiguous statements. This could lead to incorrect conclusions about the overall sentiment and themes expressed by users, thus, the interpretation of the results should be done cautiously. To mitigate these limitations, future research could expand data sources to include multiple social media platforms and online forums, conduct longitudinal studies to observe changes and trends in health information needs over time, and include non-English data to consider cultural contexts and ensure findings are inclusive and globally relevant. Additionally, complementing social media analysis with direct qualitative methods such as interviews and focus groups could provide deeper insights and validate the themes identified. By acknowledging these limitations and proposing ways to address them, we aim to provide a balanced and transparent perspective on the study’s findings and their implications for the ASD community.

## Data Availability

The raw data supporting the conclusions of this article will be made available by the authors, without undue reservation.
