# Supplementary material for: Sentiment analysis of post-COVID-19 health information needs of autism spectrum disorder community: insights from social media discussions
Source: Front Psychiatry. 2024 Oct 11;15:1441349. doi: 10.3389/fpsyt.2024.1441349 (PMC11502369; doi:10.3389/fpsyt.2024.1441349)
Supplement: Supplementary file 1 [file DataSheet1.pdf]

Supplementary Table 1. Qualitative content analysis

Theme	Representative Quotes	Analysis and Interpretation
**Symptoms**	"Every or so Wednesday I go to play DnD with my sister and some of her friends...but my ears are literally hurting at any sound and I honestly want to die so bad this is too much for me."	This quote reflects the sensory sensitivities that individuals with ASD often experience, such as hypersensitivity to sounds, which can lead to overwhelming situations and emotional distress.
	"I currently work in child care... I think she has autism spectrum disorder... She of course gets overwhelmed at the daycare center I work at when kids look or stare at her... She has difficulty with change, trouble following directions... and she often has meltdowns."	This indicates the challenges of managing symptoms like difficulty with change, sensory overload, and emotional regulation in social environments, especially for young children with ASD.
	"I have significant difficulties with loud noises and remaining still for durations of time."	Reflects the challenges faced due to sensory processing issues common in ASD, particularly with auditory stimuli and the need for movement, which can complicate medical procedures like MRIs.
**Diagnosis**	"I'm an 26yo and got my diagnosis today. I've kind of known for a while that I'm autistic but it was a pain to get confirmed...now that I know I'm not crazy and that I was right it's such a weird feeling."	Illustrates the mixed emotions individuals experience upon receiving an ASD diagnosis later in life, highlighting the difficulty of the diagnostic process and the relief of understanding one's identity.
	"I thought the diagnosis would bring me some relief and validation but if anything it's been the opposite... I keep worrying that I lied or the diagnosis is wrong."	This quote shows the uncertainty and anxiety that can accompany a diagnosis, as individuals may struggle with self-doubt or imposter syndrome even after confirmation.
	"I (26F) have both autism and ADHD which affects the way that I interact with people... I sometimes feel that it's a bit deeper due to how frequently it happens."	Reflects the intersectionality of ASD and other conditions like ADHD, and how this can complicate social interactions and self-perception.
**Caregivers of ASD**	"Our LO born at 25 weeks/85 days in NICU now 25 months just got his formal diagnosis. Needless to say we	Highlights the emotional and practical challenges faced by caregivers of children with ASD,

Supplementary Table 1. Qualitative content analysis		
**Theme**	**Representative Quotes**	**Analysis and Interpretation**
	have lots of homework & care from doctors... We are just going to make sure he has a good life and hope one day we unlock a way for him to communicate."	especially with early or severe diagnoses, and their hopes for the future.
	"Does anyone else have a lot of trouble taking care if themselves? for me i have to be woken up told when to sleep when to shower and when to eat or drink water... I'll just starve instead. I don't know why it's so hard for me."	Describes the dependency on external prompts and support often required by individuals with ASD, indicating the strain on caregivers who provide this constant support.
	"For starters I am a male 22 years old living with 'high functioning' autism... I am a person who needs consistency in my schedule to be able to take care of myself!"	Reflects the challenges faced by caregivers of young adults with ASD who require structured environments to manage daily living skills and maintain mental health.
**Caregivers' Burden**	"Every time there's a change in his routine, it's a meltdown waiting to happen. I love him, but it's so exhausting."	This quote reflects the emotional and physical exhaustion caregivers often experience when managing unpredictable behaviors and meltdowns due to ASD.
	"I'm just so tired of having to explain to everyone why he behaves the way he does...it's like a full-time job."	Highlights the social and emotional burden on caregivers who must constantly advocate and explain their loved one's behavior to others, which can be draining and isolating.
	"It feels like I have to be his voice everywhere we go. I worry what will happen when I'm not around."	This quote underscores the anxiety and long-term concerns of caregivers regarding the future independence and care of their loved ones with ASD.
**Drugs/Interventions**	"I was given a medication this year... I took the meds and they have been awful. I can't change them until after the appointment."	This quote reflects the challenges individuals with ASD face in managing medication side effects, highlighting the need for personalized medical care and flexibility in treatment plans.

Supplementary Table 1. Qualitative content analysis		
**Theme**	**Representative Quotes**	**Analysis and Interpretation**
	"The doctor offered to prescribe a pill of a benzodiazepine such as Xanax but I don't know if that would help with this or worsen it."	Indicates the uncertainty and potential risks associated with using medication to manage ASD-related symptoms, particularly in situations requiring anxiety management.
	"In the last 8 + years or so medication has helped (mostly replacing the benefits of the activity level age is now restricting) but I still am struggling just to feel accepted knowing in some ways I am but not knowing what that's actually like or how it feels or should feel."	Highlights the long-term reliance on medication to manage symptoms and the emotional complexities associated with finding effective treatments that also support overall well-being and acceptance.
**Natural Cure**	"My friend suggested trying a gluten-free diet to help with my symptoms, but I'm skeptical."	Illustrates the interest in and skepticism toward natural remedies and alternative treatments for managing ASD symptoms, highlighting a need for reliable information and guidance.
	"I've heard acupuncture might help with anxiety, but I'm not sure if it's just a placebo effect."	Shows the exploration of alternative therapies like acupuncture among individuals with ASD, often due to dissatisfaction with conventional treatments or desire for complementary approaches.
	"Someone told me meditation could help with sensory overload, but it's hard to sit still and focus."	Reflects the challenges in adopting mindfulness practices for ASD symptom management, particularly due to sensory and attention difficulties.
**Care/Treatment**	"I just wish there was a way to get personalized care...every time I see a new doctor, I have to start all over again."	This quote highlights the challenges of inconsistent medical care for individuals with ASD, emphasizing the need for personalized and continuous care.
	"The support groups help, but I feel like more could be done to actually help us navigate daily life, not just talk about it."	Indicates that while support groups provide emotional comfort, there is a need for more

Supplementary Table 1. Qualitative content analysis		
**Theme**	**Representative Quotes**	**Analysis and Interpretation**
		practical assistance and resources to manage daily living with ASD.
	"I really benefit from the occupational therapy sessions, but they’re too far and not frequent enough."	Demonstrates the value of occupational therapy for managing ASD symptoms and enhancing daily functioning, but also the barriers to accessing these services, such as location and availability.
**Stigma**	"Every time I mention my autism, people either don't believe me or they say I don't look autistic."	Highlights the stigma and misconceptions surrounding ASD, where societal expectations do not align with individual experiences, leading to invalidation and frustration.
	"I'm afraid to tell my boss about my diagnosis because I don't want to be treated differently."	Reflects the fear of discrimination and differential treatment in professional settings due to disclosing an ASD diagnosis, contributing to stress and a lack of support.
	"People often think I’m just being difficult or lazy when I can't handle loud noises or bright lights."	Indicates the misunderstanding and stigma related to sensory sensitivities in ASD, often leading to negative judgments about character or behavior.
**Smart Assistive Technologies**	"I recently got a smart watch to help remind me of tasks and routines... It’s been a game changer for me."	Shows how smart assistive technologies can significantly improve daily functioning and independence for individuals with ASD by providing reminders and structure.
	"Using a tablet with visual schedules has helped my child stay on track with their daily activities and reduced meltdowns."	Reflects the effectiveness of visual schedules provided through smart technologies in helping manage daily routines and reducing emotional distress for children with ASD.
	"The app I use helps me track my mood and sensory triggers, which has been really helpful in understanding my patterns."	Indicates the benefits of apps designed to track symptoms and triggers, enabling individuals with ASD to gain insights into

Supplementary Table 1. Qualitative content analysis		
**Theme**	**Representative Quotes**	**Analysis and Interpretation**
		their behavior and develop coping strategies.
**Burden of ASD**	"It's so hard seeing my child struggle every day and feeling like I can't do enough to help them."	This quote reflects the emotional toll on caregivers who feel helpless in alleviating the struggles of their loved ones with ASD, highlighting the need for comprehensive support systems.
	"The financial strain of therapies and treatments is overwhelming, and I worry about affording the care my child needs."	Highlights the financial burden associated with ASD care, particularly the cost of therapies and treatments, which can be a significant source of stress for families.
	"Every day feels like a battle against the world and the stigma around autism, not just the condition itself."	Reflects the dual burden faced by individuals with ASD and their caregivers, not only managing the condition but also combating societal stigma and misunderstanding.
